# Differential Responses of Soil Ammonia-oxidizing Bacterial and Archaeal Communities to Land-use Changes in Zambia

**DOI:** 10.1264/jsme2.ME24049

**Published:** 2025-03-14

**Authors:** Takamitsu Ohigashi, Suzumi Mori, Kanako Tago, Tsubasa Ohbayashi, Shintaro Hara, Yoshitaka Uchida

**Affiliations:** 1 Graduate School of Global Food Resources, Hokkaido University, 060–8589, Kita-9 Nishi-9 Kita-ku, Sapporo city, Japan; 2 Graduate School of Life Sciences, Tohoku University, 980–8578, Aramaki Aoba 6–3 Aoba-ku, Sendai city, Japan; 3 Institute for Agro-Environmental Sciences, National Agriculture and Food Research Organization, 305–8604, Kannondai 3–1–3, Tsukuba city, Japan; 4 Global Center for Food, Land and Water Resources, Research Faculty of Agriculture, Hokkaido University, 060–8589, Kita-9 Nishi-9 Kita-ku, Sapporo city, Japan

**Keywords:** land-use changes, nitrification, ammonia oxidizers, soil microbial community

## Abstract

Soil nutrient loss from intensive farming is a critical issue in sub-Saharan Africa that affects food security. While soil microbial nitrification supplies available nitrogen, excessive nitrification leads to nitrogen loss. However, the species driving nitrification and their functions in this region remain largely unknown. Therefore, we investigated the responses of ammonia-oxidizing bacterial (AOB) and archaeal (AOA) communities to land-use changes in Zambia and their relationship with nitrification potential. Soil samples were collected from three sites in Zambia that all had neighboring natural and farmed (maize) lands. We measured nitrification potential, quantified AOB and AOA, and analyzed these communities by targeting the ammonia monooxygenase subunit A (*amoA*) gene, which encodes a key enzyme in nitrification. Nitrification potential was 1.51-fold higher in farmlands than in natural lands. AOB abundance tended to be greater in farmlands, whereas AOA abundance was smaller. Farming changed the AOB community structure, increasing *Nitrosospira* cluster 3a.2 at the three sites, while minor site-specific responses were also observed. In contrast, the AOA community structure was not significantly different between land uses, but varied among sites, with cluster NS-ζ being more prominent in one site with neutral soil (pH 7.64) than in the other sites (pH 5.70 and 5.71). These results suggest that AOA species were generally vulnerable to farming, decreasing in abundance without structural changes, while some AOB species increased, driving changes in their community structure. These insights are fundamental for understanding soil nitrogen depletion due to microbial changes under farming and are crucial for developing sustainable land-use practices in sub-Saharan Africa.

Nitrogen depletion due to agricultural practices is a worldwide concern because it causes a number of environmental issues, such as soil degradation, global warming, and hydrosphere contamination (Smith *et al.*, 2008; Robertson and Vitousek, 2009). Sub-Saharan Africa, including Zambia, is one of the regions that are most severely affected by soil degradation, which has significantly impacted agricultural productivity and food security (Sanchez, 2002). One aspect of soil degradation is a decrease in soil nitrogen due to excessive farming with lower chemical input (Sanchez, 2002; Tully *et al.*, 2015; Ohigashi *et al.*, 2021). Therefore, improvements in the efficiency of nitrogen use are critical for retaining indigenous and additional nitrogen in this region (Norton and Ouyang, 2019).

In nitrogen cycling, the nitrification process involves the oxidation of ammonia/ammonium to nitrite and then to nitrate, which is the most easily lost form of nitrogen due to leaching and denitrification (Nunes-Alves, 2016; Oita *et al.*, 2016; Norton and Ouyang, 2019). The oxidation of ammonia is performed by ammonia-oxidizing bacteria (AOB) and ammonia-oxidizing archaea (AOA), and is followed by nitrite oxidation by nitrite-oxidizing bacteria (NOB) (Norton and Ouyang, 2019). The recent discovery of complete ammonia oxidation (comammox) bacteria, which solely oxidize ammonia to nitrate, adds complexity to our understanding of nitrification (Daims *et al.*, 2015; van Kessel *et al.*, 2015; Norton and Ouyang, 2019). The physiological characteristics of the two primary types of nitrifying microbes AOB and AOA, known as “ammonia oxidizers”, have been extensively exami­ned. For example, AOB favor higher pH and ammonia concentrations in soil than AOA (Prosser and Nicol, 2012; Ouyang *et al.*, 2016; Dong *et al.*, 2019; Kong *et al.*, 2019). In addition, AOA may utilize organic acids in the soil, which may play a role in detoxifying hydrogen peroxide produced during their metabolic processes, thereby supporting their persistence (Kim *et al.*, 2016; Martikainen, 2022). Furthermore, not only the general properties of AOB and AOA, but also the species included in these groups, exhibit different preferences for environmen­tal‍ ‍factors (Webster *et al.*, 2005; Tourna *et al.*, 2010; Lehtovirta-Morley *et al.*, 2016b). Therefore, investigations of the mechanisms by which soil ammonia oxidizers respond to environmental changes, such as land-use changes, provide fundamental insights into nitrogen cycling. These insights are crucial for sub-Saharan Africa, where soil nitrogen is depleted through land-use changes and there is limited information on the soil microbiome (Sanchez, 2002; Tully *et al.*, 2015; Ohigashi *et al.*, 2021; Makhalanyane *et al.*, 2023).

Previous studies demonstrated that land-use changes, including conversion to farmland, affect microbial community structures (Tardy *et al.*, 2015; de Carvalho *et al.*, 2016; Hamamoto *et al.*, 2018; Kamgan Nkuekam *et al.*, 2018; Wang *et al.*, 2018; Ohigashi *et al.*, 2021). Even in sub-Saharan Africa, the structures of prokaryotic and fungal communities have been impacted by land-use changes to farmland, including the cultivation of maize, one of the main crops in this region (Kamgan Nkuekam *et al.*, 2018; Ohigashi *et al.*, 2021). Regarding ammonia oxidizers, our previous study targeting the 16S rRNA gene showed that the relative abundance of several AOB was greater in farmlands than in natural lands (Ohigashi *et al.*, 2021), indicating a potential increase in nitrification activity in farmed soils. Although a 16S rRNA gene-based ana­lysis is useful for providing an overview of the soil microbiome, it is not sufficient for considering specific microbial functions, such as ammonia oxidation (Toole *et al.*, 2021; Wang *et al.*, 2021; Matchado *et al.*, 2024). Consequently, ammonia-oxidizing microbes have been investigated using the *amoA* gene, which encodes ammonia monooxygenase subunit A, a functional genetic marker for ammonia oxidation (Avrahami and Conrad, 2003; Alves *et al.*, 2018).

Quantification of the *amoA* gene has provided information on the overall abundance of soil ammonia oxidizers under various conditions (Hayatsu *et al.*, 2008, 2021; Carey *et al.*, 2016). In regions outside sub-Saharan Africa, the abundance of soil AOB generally increases more than that of AOA with the addition of nitrogen (*e.g.*, Carey *et al.*, 2016). However, in some acidic or nutrient-poor soils, AOA were found to be more abundant than AOB after the addition of nitrogen (Marusenko *et al.*, 2015; Wang *et al.*, 2015). Although soils in sub-Saharan Africa are generally less fertile than those in other regions and nitrogen is added during farming, our previous study demonstrated that soil pH in farmlands in Zambia remained more alkaline or neutral than in natural lands (Ohigashi *et al.*, 2021). Therefore, investigating changes in the abundance of ammonia oxidizers in nutrient-poor sub-Saharan African soils may provide novel insights into the factors regulating the niche differentiation between AOB and AOA, leading to differences in the nitrification process.

In addition to the overall abundance of ammonia oxidizers, sequencing of the *amoA* gene has provided information on their diversity at a high resolution (Dong *et al.*, 2019; Kong *et al.*, 2019; Wang *et al.*, 2019a). Previous studies showed that the addition of nitrogen changed the AOB community structure, but did not significantly affect the‍ ‍AOA community structure (Xiang *et al.*, 2017; Zhang *et‍ ‍al.*, 2018). However, limited information is currently available on how their community structures are changed by farming activities in nutrient-poor sub-Saharan Africa soil (Makhalanyane *et al.*, 2023). Since different ammonia oxidizers have varying preferences in physicochemical environments, it is important to obtain a more detailed understanding on community structural changes and responsive species to farming in nutrient-poor soils. Identifying the key nitrifying species responsible in the region may optimize the inhibition of nitrification (Nishigaya *et al.*, 2016) and prevention of nitrogen depletion.

In the present study, we assessed nitrification potential and exami­ned the abundance and community structures of AOB and AOA at three sites that had adjacent natural ecosystems and agricultural (maize) land in Zambia, sub-Saharan Africa. While previous studies demonstrated that AOA played significant roles in nitrification in acidic soils, in Zambia, soil pH remained unchanged or was higher in farmlands than in natural lands, possibly creating conditions more favorable for AOB (Ohigashi *et al.*, 2021). Therefore, we hypothesized the following: (1) due to farming, the abundance of AOB may increase more than that of AOA; (2) the community structure of AOB is more likely to change than AOA, even in Zambian nutrient-poor soil; and (3) these microbial changes may be related to differences in nitrification potential. The present results will contribute to a more detailed understanding of the micro-scale mechanisms of nitrogen loss due to agriculture in sub-Saharan Africa.

## Materials and Methods

### Sampled soils

In January 2019, soil sampling was conducted in Lusaka and the Central Province in Zambia. The climate of this region is sub-tropical with an annual rainfall of approximately 893‍ ‍mm (Data Africa, accessed on July 28, 2024: https://dataafrica.io/profile/zambia). The sampling locations, Site A (15°32′S. 28°15′E.), Site B (14°39′S. 28°02′E.), and Site C (14°23′S. 28°29′E.), were selected from three pairs of neighboring natural and farmed lands that were approximately 100‍ ‍m apart. The vegetation in natural lands was bush, considered to be miombo woodlands typical in this region (Pelletier *et al.*, 2019), while maize was the major crop in farmlands. Approximately 100‍ ‍g of soil was taken from the near surface (0 to 10‍ ‍cm) for sampling, with three replications for each land use at each site. These replications were located approximately 20‍ ‍m apart. The locations of the sample sites were recorded using a GPS logger (eTrex 20; Garmin). Sampled soils were imported to Japan following the guidance of the Yokohama Plant Protection Station within 8 days. Samples were then stored at room temperature for further ana­lyses. We avoided refrigeration because it may reduce nitrification activity in soils (Verchot, 1999).

According to local farmers, the farmlands at Sites A, B, and C had been under cultivation for 2, 4, and 54 years, respectively, up to the time of sampling. Urea (nitrogen: 46%) was applied annually to the farmed sites at rates of 100–200, 193, and 200‍ ‍kg ha^–1^ at Sites A, B, and C, respectively. Additionally, a common basal fertilizer in sub-Saharan Africa called Compound D (nitrogen: 10%, phosphorus: 20%, potassium: 10%) was used to supplement soil nutrients at rates of 100–200, 97, and 250‍ ‍kg ha^–1^ at Sites A, B, and C, respectively. Other management practices, including the application of organic matter, crop rotations, and tillage styles are summarized in Table S1.

A previous study (Ohigashi *et al.*, 2021) reported the fundamental properties of the soils investigated herein. In brief, soil textures were classified as sandy loam at Sites A and C and sandy clay loam at Site B. According to GPS data and the Soil Atlas of Africa (Dewitte *et al.*, 2013) exami­ned using QGIS version 3.20.1, the soil type was identified as Chromic Lubisols at Site A and Undifferentiated Acrisols at Sites B and C. Averaging the two land uses (natural and farm) within each site, soil pH was higher at Site A (pH 7.64±0.52) than at Site B (pH 5.70±0.41) and Site C (pH 5.71±0.40), whereas farmed soils at Site A (pH 8.05±0.37) and Site C (pH 5.90±0.41) had a higher pH than their counterpart natural soils (pH 7.23±0.25 and pH 5.52±0.27, respectively). The total carbon content was higher in natural soils (1.72±0.54%) than in farmed soils (1.23±0.60%). Similarly, the total nitrogen content was higher in natural soils (0.24±0.08%) than in farmed soils (0.16±0.08%).

### Measurements of inorganic nitrogen and nitrification potential

Sampled soils were mixed inside storage bags for homogenization. Five grams of soil samples from each replication was shaken with 50‍ ‍mL of 10% KCl for 1 h, and the mixture was then filtered through filter paper (Grade 5C, <5‍ ‍mm; Advantec). Filtered extracts were stored at –30°C until further ana­lyses. NH_4_^+^-N and NO_3_^–^-N concentrations were assessed using a colorimetric method with a flow injection analyzer (AQLA-700; Aqualab) as previously described (Oka and Uchida, 2018).

To measure nitrification potential, an incubation was conducted using the shaken-slurry method (Hart *et al.*, 1994). In each replication, 15‍ ‍g of sampled soil was mixed with 100‍ ‍mL of 0.75‍ ‍mM ammonium sulfate, which was adjusted to pH 7.2 to eliminate the effects of pH on nitrification during the incubation. Soil slurries were placed in 300-mL Erlenmeyer flasks and incubated at room temperature while being shaken at 50‍ ‍rpm until the complete conversion of ammonium to nitrate was achieved. Complete conversion was indicated by the absence of detectable ammonium in periodically collected slurries in the following steps. One hour after the start of the incubation, a 1.5-mL sample of the slurry was collected for DNA extraction to obtain DNA of the ammonia oxidizers facilitating nitrification being investigated in the present study. Additionally, during the incubation, 10‍ ‍mL of the slurry was periodically collected. The timing of sampling for each replication was adjusted based on the progress of nitrification, as measured by the inorganic nitrogen concentration of the slurry. The specific timing of sampling is shown in Table S2. To measure inorganic nitrogen (NH_4_^+^-N and NO_3_^–^-N), a 10-mL sample of the slurry was shaken with 30‍ ‍mL of 10% KCl at 200‍ ‍rpm for 1 h. The mixture was then subjected to the same method to measure inorganic nitrogen concentrations using a flow injection analyzer, as described above. The nitrate concentration showed a linear increase over time, allowing the calculation of nitrification potential (mg NO_3_^–^-N‍ ‍kg^–1^‍ ‍h^–1^) based on the rate of the nitrate concentration increase within the slurry using linear regression.

### DNA extraction and quantitative PCR

Soil DNA was extracted from 1.5‍ ‍mL of slurry taken 1‍ ‍h after the incubation had started using NucleoSpin^®^ Soil (Macherey-Nagel GmbH & Co. KG) according to the protocol provided by the manufacturer. Extracted DNA was stored at –30°C until further ana­lyses. The copy numbers of the AOB and AOA *amoA* genes were quantified by an SYBR Green-based qPCR technique using a Mx3005P (Agilent Technologies) with KAPA SYBR (Agilent). Primer sets and qPCR conditions were selected according to previous studies (Meinhardt *et al.*, 2015; Gao *et al.*, 2017). To quantify the total abundance of the AOB *amoA* gene, amoA-1Fmod (5′-CTGGGGTTTCTACTGGTGGTC-3′) and GenAOBR (5′-GCAGTGATCATCCAGTTGCG-3′) with amplicon lengths of 120 bp were used. Twenty-five microliters of the mixture, which contained 12.5‍ ‍μL KAPA SYBR (Agilent), 0.5‍ ‍μL of each AOB *amoA* gene primer, 2‍ ‍μL of diluted DNA, and 9.5‍ ‍μL of nuclease-free water, was amplified under the following conditions: at 95°C for 10‍ ‍min, followed by 40 cycles at 95°C for 30‍ ‍s, 58°C for 1‍ ‍min, and 72°C for 1‍ ‍min. To quantify the total abundance of the AOA *amoA* gene, GenAOAF (5′-ATAGAGCCTCAAGTAGGAAAGTTCTA-3′) and GenAOAR (5′-CCAAGCGGCCATCCAGCTGTATGTCC-3′) with amplicon lengths of 134 bp were used. Mixture and cycle conditions were the same as those for the AOB *amoA* gene, except for the annealing temperature (56°C). The threshold line was calculated by R (version 3.6.3), and the result was shown as copy numbers. In each ana­lysis, a melting curve ana­lysis was performed to check for the specificity of the amplified PCR product. To quantify gene copy numbers, standard curves were generated from a 10-fold serial dilution of the amplicons containing the target gene fragments, which were preliminarily selected by the DNA concentration measured with Quantus (Promega Corporation). The efficiency of qPCR was in the range of 83.9–103.1%.

### Microbial DNA sequencing

Extracted DNA samples were amplified by PCR. In PCR of AOB *amoA*, the forward primer amoA-1Fmod labeled with the Ion Xpress Barcode Adapters Kit (Thermo Fisher Scientific) and the reverse primer GenAOBR labeled with the Ion P1 adapter (Thermo Fisher Scientific) were used. The mixture, which contained 2‍ ‍μL of extracted DNA, was mixed with 12.5‍ ‍μL of Amplitaq Gold Master Mix (Applied Biosystems^TM^), 0.5‍ ‍μL of each primer, and 9.5‍ ‍μL of nuclease-free water and amplified under the following cycle conditions: at 95°C for 10‍ ‍min, followed by 40 cycles at 95°C for 30‍ ‍s, 58°C for 1‍ ‍min, and 72°C for 1‍ ‍min. In PCR of the AOA *amoA* gene, mixture and cycle conditions were the same as those for the AOB *amoA* gene, except for the primers (the labeled forward primer GenAOAF and the labeled reverse primer GenAOAR) and annealing temperature (56°C). All PCR products obtained were then purified with AMPure XP (Beckman Coulter) according to the manufacturer’s protocol. The molarity of the amplicons was checked by Bioanalyzer 2100 (Agilent) using the Bioanalyzer High Sensitivity DNA Kit (Agilent) according to the manufacturer’s protocol. The libraries were then diluted to 50 pM using nuclease-free water. The Ion Chef Instrument (Thermo Fisher Scientific) with the Ion PGM Hi-Q Chef kit was used to load the library into the Ion 318 chip (Thermo Fisher Scientific). DNA sequencing was performed on the Ion PGM Sequencer (Thermo Fisher Scientific) with Ion PGM 400 kits. The DNA sequence data used in the present study can be accessed on NCBI with the accession numbers PRJNA985890 for AOB and PRJNA985886 for AOA.

To establish whether incubation conditions adequately reflected the original state of the microbial community in the field, 16S rRNA gene amplicons from the slurry after an incubation for 1‍ ‍h were subjected to DNA sequencing, an ana­lysis of diversity, and comparisons with the microbial community in the environment, which ensured consistency (Fig. S4). We assessed ammonia oxidizer communities at both the 1-h and end-of-incubation points, and confirmed no significant shifts in community compositions between these two time points (Fig. S5). The method is described in detail in the Supplementary materials.

### Analysis of sequenced DNA

Sequence data were obtained from the Ion Torrent system and analyzed using the dada2 package on R software (3.6.3) for adapter trimming, quality filtering, denoising, chimera removal, and identification of amplicon sequencing variants (ASVs) (Callahan *et al.*, 2016). In adapter trimming, the sequences obtained were truncated by detecting the primers of AOB and AOA using the cutadapt command. The sampling depth for random subsampling to equalize sample sizes was the lowest number of non-chimeric sequences among the samples, which were 1,616 and 8,370 for AOB and AOA, respectively (Table S3). Based on the sequences, we obtained 160 and 403 ASVs for AOB and AOA, respectively. To extract ASVs phylogenetically close to isolated and cultured ammonia oxidizers, reference sequence databases (https://github.com/taka-ohi/zambian_ammonia_oxidizers/) were created using the makeblastdb function in ncbi-blast+ (2.9.0). ASVs that showed similarity to the reference sequences were extracted using the blastn function in ncbi-blast+, employing an E value threshold <10. As a result, 133 ASVs and 25 ASVs that had 84.8–100.0 and 81.5–100.0% similarities to the references for AOB and AOA, respectively, were extracted and used in subsequent phylogenetic and community-structural ana­lyses. Regarding each of the AOB and AOA communities, a phylogenetic tree of ASVs that had an average total abundance >1.0% in all groups was created in MEGA X (Kumar *et al.*, 2018) after sequences were aligned with the above reference sequences using the MUSCLE algorithm (Edgar, 2004). Maximum likelihood phylogenetic trees were constructed with 1,000 bootstrap replicates using the Tamura-Nei nucleotide substitution model in MEGA X. Phylogenetic trees were then plotted using iTOL v5 (Letunic and Bork, 2021). Clusters of AOB and AOA in phylogenetic trees were identified and labeled based on the positions of the reference sequences in the trees, referring to previous studies that provided phylogenetic trees for AOB (Avrahami and Conrad, 2003; Yang *et al.*, 2017; Wang *et al.*, 2019a) and AOA (Alves *et al.*, 2018).

### Statistical ana­lysis

A two-way ana­lysis of variance (ANOVA) was performed to assess variations in the inorganic nitrogen content, nitrification potential, and copy numbers for AOB and AOA, considering the factor of land use (natural or farm) and site (Site A, Site B, or Site C). Prior to conducting the two-way ANOVA, the normality and homogeneity of variance were assessed using the Shapiro-Wilk test in R. If *P*-values for the test were lower than 0.05, data were transformed using logarithmic transformation and subsequently analyzed using the two-way ANOVA. Dissimilarities in the community structures of AOB and AOA were analyzed through non-metric multidimensional scaling (NMDS), employing the Bray-Curtis dissimilarity index. This ana­lysis was conducted using the metaMDS function from the vegan package (Dixon, 2003) in R. To assess differences in community structures between land-use types, a permutational multivariate ana­lysis of variance (PERMANOVA) was employed. This ana­lysis considered the factors of ‘land use’ (Farm=1, Natural=2) and the factor ‘site’ (A=1, B=2, C=3). The adonis function from the vegan package was used for this ana­lysis. Clusters of ammonia oxidizers and the following environmental factors: total nitrogen, total carbon, and pH, measured by Ohigashi *et al.* (2021), and NH_4_^+^-N and NO_3_^–^-N contents measured in the present study were plotted if the relative abundance of clusters or environmental factors correlated with community structures (*P*<0.05), which was exami­ned using the envfit function in the vegan package. To examine differences in the relative abundance of ASVs between land uses within a site, the ANCOM test was conducted using the ancombc function in R (Lin and Peddada, 2020). Heatmaps showing the scaled relative abundance of AOB and AOA were generated using the heatmap.2 function in R. Prior to plotting, relative abundance was scaled using the scale function in R as having a mean of zero and standard deviation of one. Spearman’s correlation test was conducted to estimate the contributions of each AOB/AOA cluster, their overall abundance, and environmental variables to nitrification potential. In this test, the estimated absolute abundance of the AOB and AOA clusters was calculated using relative abundance and qPCR data, as previously described (Jian *et al.*, 2020).

## Results

### Inorganic nitrogen contents and nitrification potential of soils

Significant differences were observed in the original ammonia content in soils among the sites (Two-way ANOVA, *P*<0.05), but not in the nitrate content across the sites and land-use changes (Fig. S1).

**Fig. 1.**
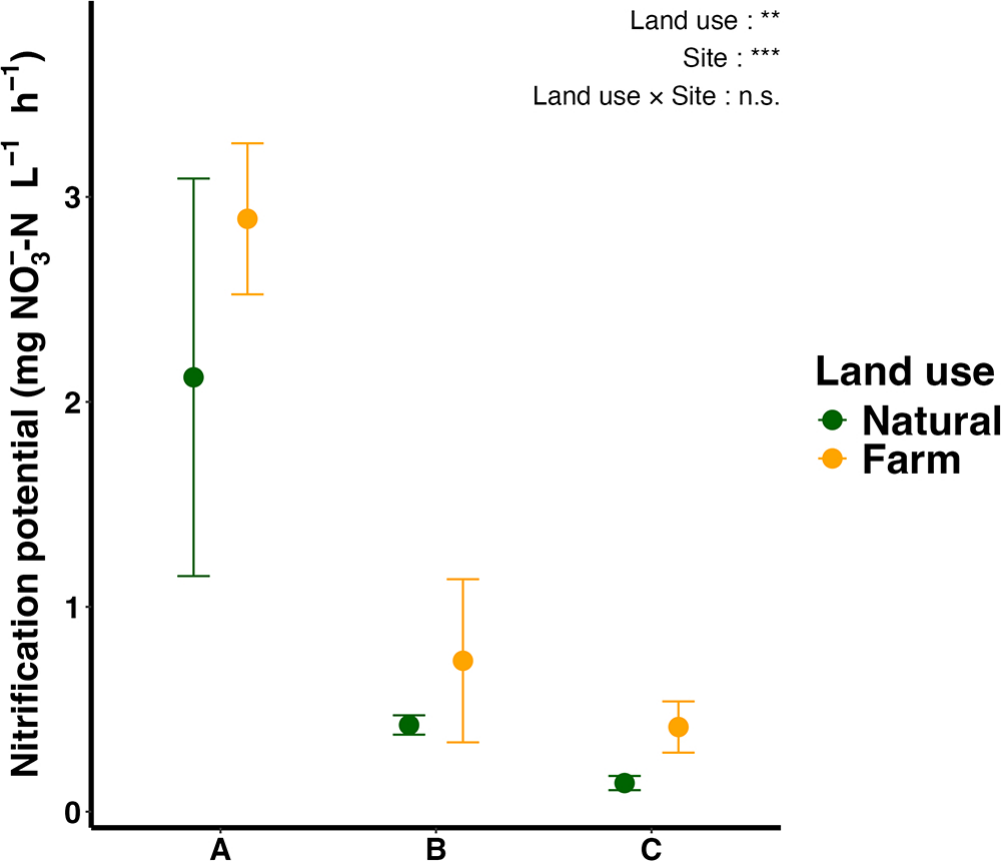
The nitrification potential of soils. Points represent mean values and error bars represent standard deviations. Data were subjected to a two-way ANOVA for the site and land use after logarithmic transformation due to non-normality, which were tested using the Shapiro-Wilk test. The significance of differences in effects are shown with *, **, or ***, representing *P*<0.05, *P*<0.01, or *P*<0.001, respectively.

Nitrification potential significantly differed among the sites (*P*<0.05), as indicated by the two-way ANOVA of logarithmically transformed values (Fig. 1). On average, the nitrification potential was 2.51±0.71‍ ‍mg NO_3_^–^-N L^–1^ h^–1^ at Site A, which was approximately 4.33- and 8.96-fold higher than that at Site B (0.58±0.28‍ ‍mg NO_3_^–^-N L^–1^ h^–1^) and Site C (0.28±0.16‍ ‍mg NO_3_^–^-N L^–1^ h^–1^), respectively. Additionally, nitrification potential was approximately 1.51-fold higher in farmed soils than in natural soils, considering all sites on average (Two-way ANOVA, *P*<0.05).

### Abundance of AOB and AOA

**Fig. 2.**
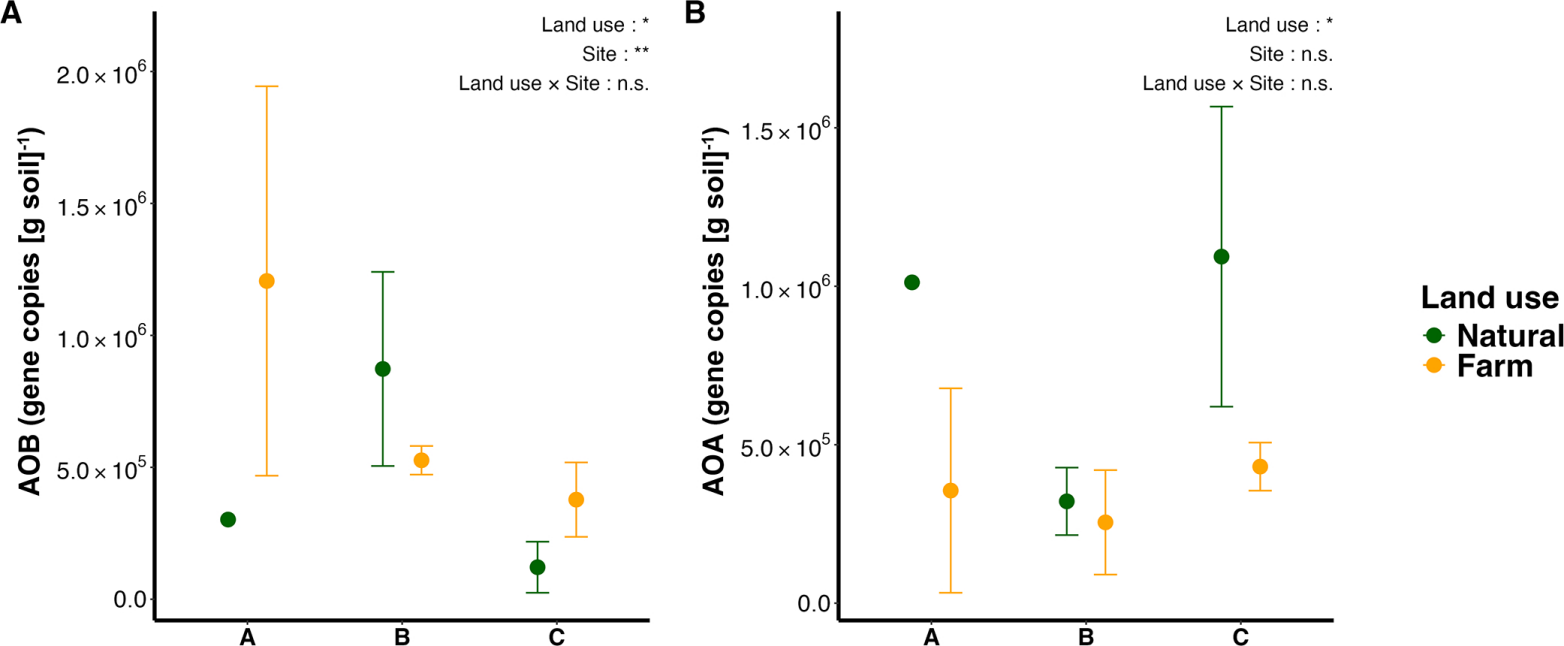
The abundance of AOB and AOA. The mean copy numbers (g dry soil)^–1^ of AOB (A) and AOA (B) are shown as points. Error bars represent standard deviations. Data were log-transformed and analyzed with a two-way ANOVA for the site and land use. The significance of differences in effects are shown with *, **, or ***, representing *P*<0.05, *P*<0.01, or *P*<0.001, respectively.

In the present study, the copy number of AOB (g^–1^ dry soil) ranged between 4.21×10^4^ and 1.84×10^6^, while that of AOA ranged between 8.87×10^4^ and 1.40×10^6^. The abundance of AOB significantly varied among the sites and showed a significant 1.62-fold increase due to land-use changes to farmlands (Two-way ANOVA, *P*<0.05), whereas a slight decrease was observed in farmed soil at Site B (Fig. 2A). In consideration of the average across all sites, the abundance of AOA was significantly higher (2.33-fold) in natural soils than in farmed soils (Two-way ANOVA, *P*<0.05) (Fig. 2B).

### Phylogenetic trees of AOB and AOA

**Fig. 3.**
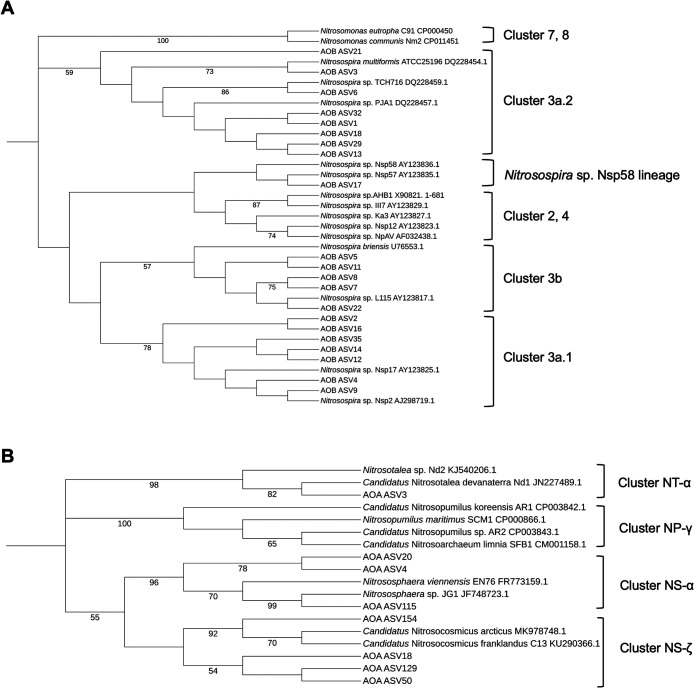
Phylogenetic trees of AOB and AOA. The phylogenetic trees of (A) AOB and (B) AOA are shown. ASVs accounting for >1.0% on average in all samples and reference sequences were used for trees. Trees were constructed based on *amoA* gene sequences using the maximum likelihood method with 1,000 iterations. Bootstrap values (>50) are indicated at branch points. Branch lengths are ignored.

In the AOB community (ASVs harboring >1.0% of abundance in all samples regardless of treatments), all ASVs were affiliated with the genus *Nitrosospira* (Fig. 3A). The relative abundance of each cluster in relation to overall abundance across all treatments was as follows: cluster 3a.1, 29.8%; cluster 3a.2, 37.6%; cluster 3b, 15.1%; *Nitrosospira* sp. Nsp58 lineage, 1.85%; and Others (unassigned or <1.0%), 15.7%. Regarding the AOA community (ASVs harboring >1.0% of abundance in all samples regardless of treatments), the majority of ASVs were affiliated with the genus *Nitrososphaera* (NS-α and NS-ζ), while one ASV showed similarity to the genus *Nitrosotalea* (NT-α) (Fig. 3B). The relative abundance of each cluster in relation to overall abundance across all treatments was as follows: cluster NT-α, 21.8%; NS-α, 43.2%; NS-ζ, 32.7%; and Others (unassigned or <1.0%), 2.27%.

### Community structures of AOB and AOA, and their relationship with environmental factors and nitrification potential

**Fig. 4.**
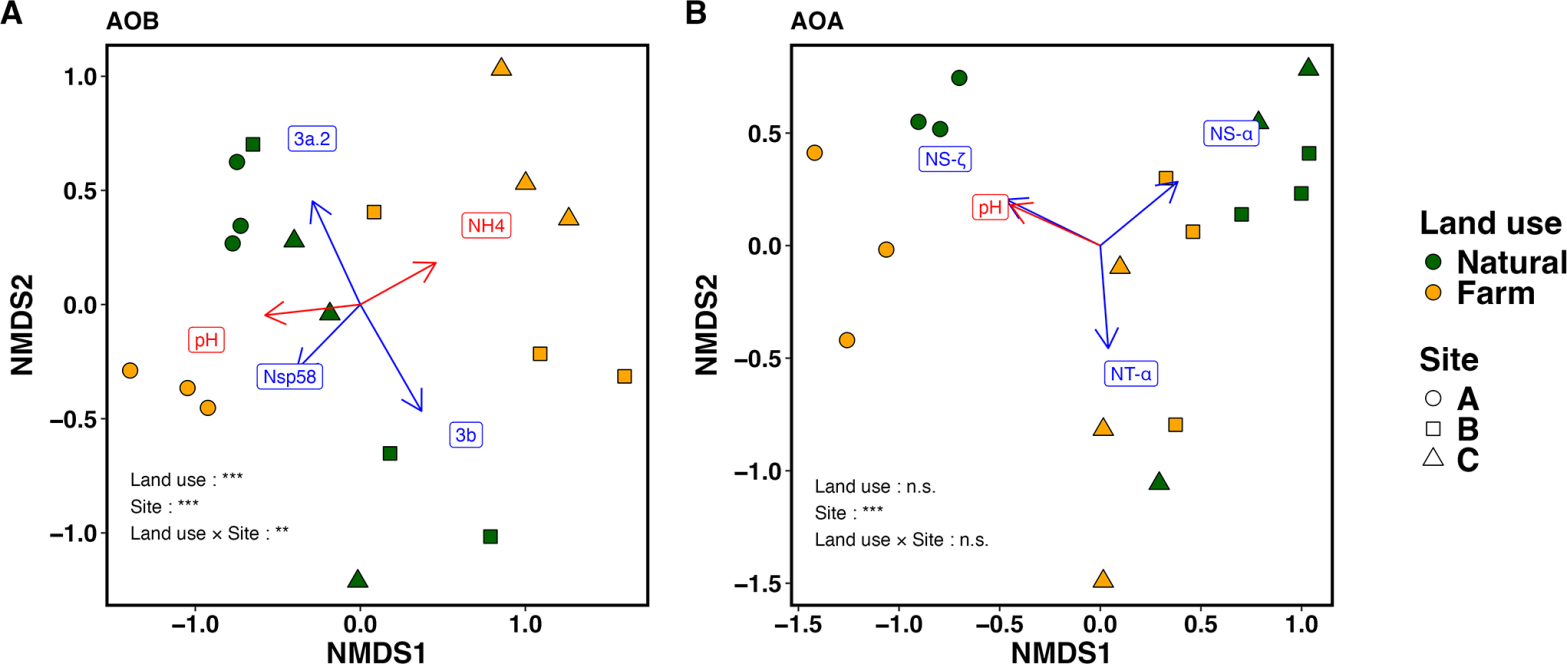
Community structures of AOB and AOA. Non-metric multidimensional scaling plots for (A) AOB and (B) AOA communities are shown. The effects of site and land-use changes were tested with PERMANOVA. The significance of differences in effects are shown with *, **, or ***, representing *P*<0.05, *P*<0.01, or *P*<0.001, respectively. Clusters and environmental factors with correlations (*P*<0.05) between the communities are shown in blue and red, respectively.

**Fig. 5.**
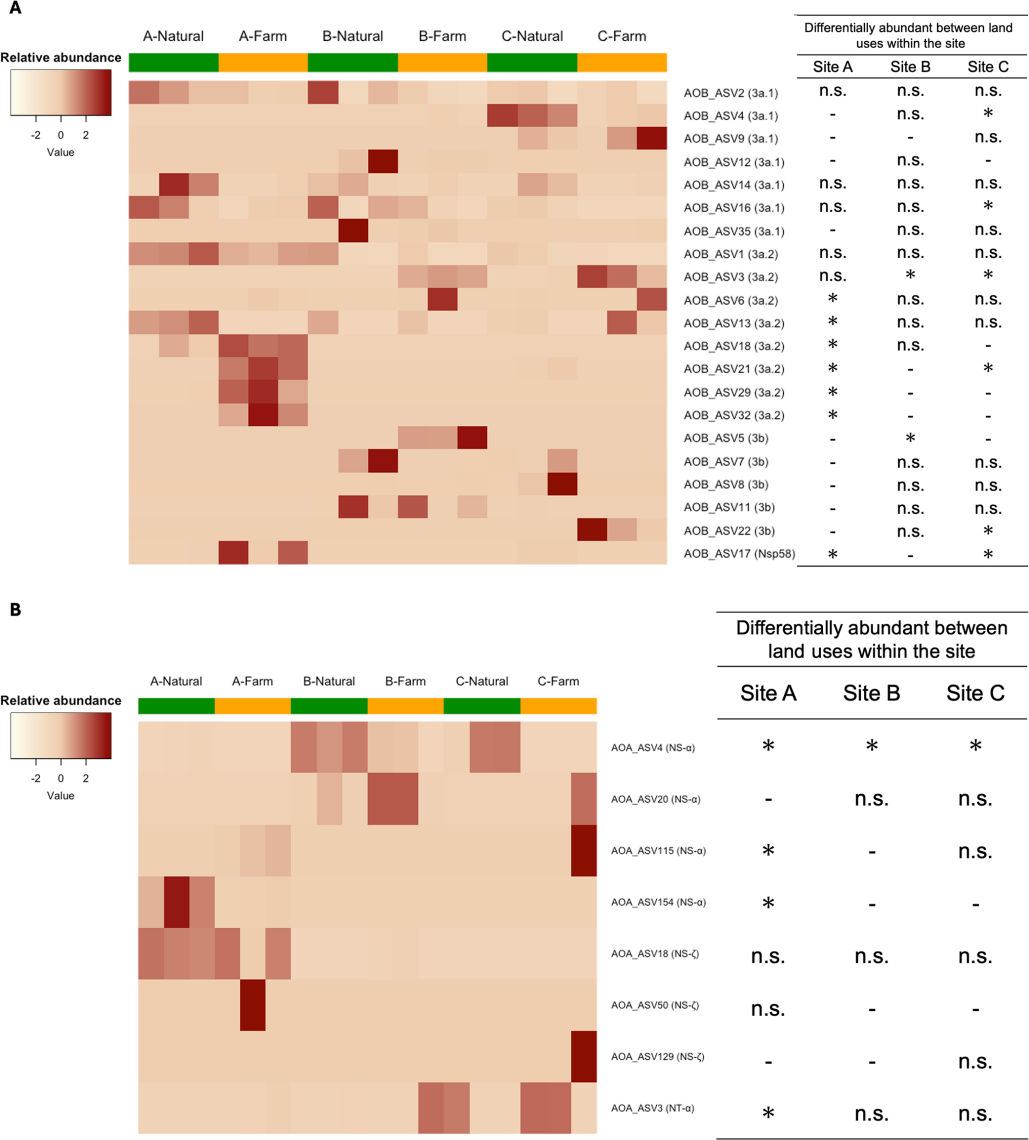
Distribution of ASVs and effects of land-use changes on their abundance. Heatmaps of the scaled relative abundance of (A) AOB-ASVs and (B) AOA-ASVs accounting for >1.0% of total abundance were plotted. Clusters of ASVs are indicated on the right side of their names. The results of differential abundance tests with ANCOM are shown in the table. Differentially or commonly abundant ASVs between land uses within each site are indicated by “*” or “n.s. ”, respectively. No testing was performed to compare land uses for ASVs that were not observed in any sample within six samples in each site, which were indicated as “-”.

Differences in the community structures of AOB and AOA and the clusters that correlated with these structures are shown in Fig. 4. A significant interaction was observed between land use and site in AOB community structures (PERMANOVA, *P*<0.05), indicating that the effects of land-use conversion on community structures depended on the site (Fig. 4A). The site-specific effects of land-use changes were evident, with a positive correlation being observed between the Nsp58 lineage and farmland at Site A. While the relative abundance of cluster 3b did not strongly correlate with the farmland community (Fig. 4A), the relative abundance of specific ASVs within this cluster, AOB_ASV5 and AOB_ASV22, was significantly higher in farmlands at Sites B and C, respectively (Fig. 5A; ANCOM, *P*<0.05). In addition to the site-specific effects of land-use changes, consistent patterns were observed across all sites; the relative abundance of ASVs in cluster 3a.2 was slightly higher in farmlands than in natural lands (Fig. 5A; ANCOM, *P*<0.05). Moreover, the relative abundance of cluster 3a.1 remained unchanged or became lower in farmlands (Fig. 5A and S2A). Regarding the relationship between the AOB community and environmental factors, the ammonia content positively correlated with the farmland communities at Sites B and C, whereas soil pH positively correlated with the community at Site A (Fig. 4A).

The AOA community structure was significantly characterized by the site (PERMANOVA, *P*<0.05), with predominant clusters varying across sites, including cluster NS-ζ at 90.4% at Site A, cluster NS-α at 77.5% at Site B, and cluster NT-α at 47.5% at Site C (Fig. S2B). Nevertheless, a consistent effect of land-use changes across sites was still observed at the ASV level; a lower relative abundance of AOA_ASV4 (NS-α) in farmlands than in natural lands was observed at all sites (Fig. 5B; ANCOM, *P*<0.05). Regarding the relationship between AOA communities and environmental factors, soil pH strongly correlated with the community at Site A (Fig. 4B).

Nitrification potential positively correlated with soil pH and the estimated absolute abundance of clusters 3a.2 and NS-ζ (Fig. S3; Spearman’s test, *P*<0.05). In contrast, the total abundance of ammonia oxidizers did not correlate with nitrification potential (Fig. S3).

## Discussion

In the present study, the effects of land-use changes on AOB community structures varied among sites, with soil pH and ammonia content correlating with these changes (Fig. 4A). This study identified two key patterns: (1) a consistent phenomenon across all sites where land-use changes led to a relative decrease in cluster 3a.1 and a relative increase in cluster 3a.2, and (2) the site-specific effects of land-use changes, characterized by a relative increase in the Nsp58 lineage at Site A and a relative increase in cluster 3b at Sites B and C (Fig. 5A and S2A).

By focusing on changes in the AOB community due to nitrogen fertilization associated with farming, (1) the consistent turnover between clusters 3a.1 and 3a.2 across all sites was in agreement with the findings of a previous study conducted in the monsoon region of China (Zhong *et al.*, 2016). In that study, nitrogen fertilization rates of between 0 and 60% led to an increase in cluster 3a.2, while rates of between 80 and 100% caused a slight decrease (Zhong *et al.*, 2016; Supplementary data). In contrast, in other studies conducted in China using stable isotope labeling methods on soils supplied with urea or ammonia, 78.9–98% of active AOB were cluster 3a.1 (Kong *et al.*, 2019; Wang *et al.*, 2019a). These differences in AOB community responses to nitrogen fertilization may be explained by pH changes that occur following the application of nitrogen (or farming practices). In the study where cluster 3a.1 became dominant following nitrogen fertilization, soil pH decreased from 7.25±0.03 in the unfertilized group to 5.86±0.05 in the chemical fertilization group (Kong *et al.*, 2019). Conversely, in the present study where cluster 3a.2 became dominant in farmlands, soil pH was higher in farmlands at Sites A and C than in their counterpart natural lands (Site A: 7.23±0.25 to 8.04±0.37, Site C: 5.52±0.27 to 5.90±0.41) (Ohigashi *et al.*, 2021). Similarly, in the study that observed an increase in cluster 3a.2, soil pH remained relatively unchanged (nitrogen fertilization rate 0%: 6.34±0.07, 40%: 6.31±0.09, and 60%: 5.67±0.34) (Zhong *et al.*, 2016). This preference of cluster 3a.2 for higher pH may be explained by the optimal growth condition for *Nitrosospira multiformis*, a member of this cluster, which has an optimal pH of 7.5 but does not grow at pH below 6.0 or above 8.2 (Watson *et al.*, 1971). Soil pH was previously proposed as the primary factor affecting the AOB community structure in a sugarcane field (Tago *et al.*, 2015). The present results indicate that soil pH also played a critical role in the relative increase observed in cluster 3a.1 or 3a.2 following farming practices in Zambia. Although there was no significant difference, nitrate content, a contributor to soil acidity, was relatively lower in farmlands than in natural lands (Fig. S1B, D), which may have led to the higher soil pH in farmlands (Shi *et al.*, 2009).

On the other hand, there were (2) site-specific effects of land-use changes on the AOB community, represented by the relative increase in the Nsp58 lineage in the farmland at Site A and the relative increase in cluster 3b in the farmlands at Sites B and C. In a semi-arid farmland soil in Inner Mongolia, the Nsp58 lineage occurred with a high pH of 8.11±0.03 (Chen *et al.*, 2015), which corresponds to the pH range in the farmland soil at Site A (8.04±0.37). The increased pH in the farmland at Site A may also favor this lineage in the present study. Moreover, culture-based studies showed that *Nitrosospira briensis* and *Nitrosospira* sp. L115 from cluster 3b continued to grow under high-ammonia conditions where other AOB clusters cannot survive (Webster *et al.*, 2005; Tourna *et al.*, 2010). The ammonia content was slightly higher in the farmlands at Sites B and C than in their counterpart natural lands, but was slightly lower at Site A (Fig. S1A). This may be attributed to Sites B and C obtaining additional ammonia through the application of organic matter in addition to chemical fertilizers (Table S1). At Sites B and C, where pH was originally lower and the ammonia content was higher, the microbial community may have been more specified for the environment (Nicol *et al.*, 2008; Prosser and Nicol, 2012). As observed in the natural lands at Sites B and C (Fig. S2A), species in cluster 3b may have been among the selected microbes. The further relative increase in ammonia in the farmlands at these sites may have created a more favorable environment for this cluster, possibly leading to its increase due to a priority effect (Debray *et al.*, 2022). Alternatively, the effects of organic matter itself, not the ammonia content, may drive the site-specific relative change in the AOB community through land-use changes (Tao *et al.*, 2021). However, this requires further investigations to elucidate the underlying mechanisms. Collectively, these findings suggest that the site-specific effects of land-use changes on the AOB community may be affected by initial differences in the site’s soil characteristics, such as pH and ammonia content, and further modifications through the effects of farming practices. Despite these relative changes in the abundance of the Nsp58 lineage and cluster 3b, the estimated absolute abundance of these clusters did not positively correlate with nitrification potential (Fig. S3). Therefore, the site-dependent effects of land-use changes may be insufficient to significantly contribute to nitrification potential.

In contrast to AOB, the AOA community structure was more strongly affected by the sites than by land-use changes, showing a correlation with soil pH (Fig. 4B). Regarding the effects of nitrogen fertilization during farming, previous studies also indicated that AOA community structures remained relatively unchanged in response to the addition of nitrogen, unlike AOB community structures (Xiang *et al.*, 2017; Zhang *et al.*, 2018). Besides, soil types (*e.g.*, Lubisol and Acrisol) have been suggested to play a more crucial role in forming the AOA community structure than farming practices (Chen *et al.*, 2010; Xu *et al.*, 2012). The present study provides further evidence for the robustness of AOA community structures to land-use changes, even in Zambia. However, it is important to note that while the AOA community structure was not markedly affected by land-use changes (Fig. 4B), the abundance of AOA was significantly decreased (Fig. 2B). The results obtained herein suggest that while the structural composition of the AOA community remained robust under farming conditions, overall abundance was more vulnerable, leading to its decrease without significant shifts in the community composition. This vulnerability may be due to the relatively increased soil pH, exposure to ammonia by fertilization, and the potential decrease in organic acids resulting from reduced soil carbon (Prosser and Nicol, 2012; Kim *et al.*, 2016; Ohigashi *et al.*, 2021). However, the overall abundance of AOA did not correlate with these environmental factors (Fig. S3), indicating that no single factor fully explains the impact of land-use changes on AOA.

Regarding the site-dependent characteristics of the AOA community structure, cluster NS-ζ was distinctive at Site A, while clusters NS-α and NT-α were prominent at Sites B and C (Fig. 4B and S2B). The relative abundance of cluster NS-ζ strongly correlated with soil pH (Fig. 4B and S3). This positive correlation is consistent with a previous study conducted in Germany, although it was 16S rRNA-based, but linked with the *amoA* gene by constructing a database using published genomes (Wang *et al.*, 2021). *Candidatus* Nitrosocosmicus franklandus C13, a member of cluster NS-ζ, was originally isolated from sandy loam arable soil with a pH of 7.5 in Scotland and exhibited higher resistance to ammonium and nitrite concentrations than other neutrophilic AOA (Lehtovirta-Morley *et al.*, 2016a). Although the soil at Site A did not contain highly concentrated inorganic nitrogen, the high pH (pH 7.64±0.52 on average) may have been advantageous for AOA in this cluster. Our results on nutrient-poor soil showed that cluster NS-ζ preferred high pH conditions, most likely independent of soil inorganic nitrogen levels. On the other hand, cluster NT-α at Sites B and C exhibited distinct characteristics from that at Site A (Fig. 4B and S2B). *Nitrosotalea* sp. Nd2 within cluster NT-α was previously reported to be abundant in acidic soils with a pH of approximately 5.0 (Prosser and Nicol, 2016), and *Candidatus* Nitrosotalea devanaterra, also within cluster NT-α, grew in cultures within a pH range of 4.0 and 5.5 (Lehtovirta-Morley *et al.*, 2016b). Furthermore, a previous study on wetland soils found a negative correlation between the abundance of NT-α and soil pH, indicating a preference for acidic soils (Wang *et al.*, 2021). Therefore, the lower soil pH at Sites B and C than at Site A may have provided favorable conditions for this cluster in the present study. However, the increase in soil pH within Site C resulting from farming may not have been sufficient to decrease the relative abundance of this cluster (Fig. S2B). This robustness suggests that the AOA community structure is primarily changed by site dependencies rather than by land uses (Fig. 4B).

Although the AOA community structure was strongly dependent on the site, at the ASV level, AOA_ASV4 belonging to cluster NS-α was decreased by farming common to all sites (Fig. 5B). *Nitrososphaera viennensis*-like OTUs (“NS-α” according to Alves *et al.*, 2018) decrease with the addition of nitrogen (Dong *et al.*, 2019). In a culture-based study, *N. viennensis* grew at 1 to 15‍ ‍mM NH_4_^+^, but not at 20‍ ‍mM NH_4_^+^ (Tourna *et al.*, 2011). Therefore, fertilization in farms may suppress the growth of AOA in cluster NS-α, leading to a relative decrease in AOA_ASV4 (NS-α) in farmed soils.

In addition to the relative abundance of AOB and AOA, it is important to consider their overall abundance because numerous studies showed their contribution to nitrification potential (Jia and Conrad, 2009; Xia *et al.*, 2011; Tago *et al.*, 2015). In the present study, the copy number of AOB (g^–1^ dry soil) ranged between 4.21×10^4^ and 1.84×10^6^, while that of AOA ranged between 8.87×10^4^ and 1.40×10^6^ (Fig. 2). These values were lower than those in the majority of soils investigated in 14 other studies conducted in different regions (Table S4). The lower abundance of ammonia oxidizers may‍ ‍be related to nutrient-poor conditions in sub-Saharan African soils (Sanchez, 2002; Tully *et al.*, 2015; Ohigashi *et al.*, 2021). Based on the average across the sites tested, the abundance of AOB increased with farming while that of AOA decreased (Fig. 2). Therefore, the abundance of AOB was greater than that of AOA in farmed soils at all sites. Previous studies reported that the addition of nitrogen to soil increased the abundance of AOB more than that of AOA (Prosser and Nicol, 2012; Carey *et al.*, 2016; Ouyang *et al.*, 2016; Xiang *et al.*, 2017; Dong *et al.*, 2019; Kong *et al.*, 2019). AOB generally prefers environments with higher ammonia concentrations more than AOA, which is attributed to their lower affinity for ammonium than AOA (He *et al.*, 2012; Prosser and Nicol, 2012). The stimulation of AOB by urea fertilization may be one of the reasons for the greater abundance of AOB in farmed soils in this study, even though the ammonium content did not show a significant increase with land-use changes (Fig. S1A). However, other studies demonstrated a shift towards AOA dominance over AOB following the addition of nitrogen in nutrient-poor soils with limited available nitrogen (Wang *et al.*, 2015), as well as in desert soils (Marusenko *et al.*, 2015). However, in these soils, the addition of chemical nitrogen fertilizers led to a decrease in soil pH, creating a niche that is favorable to AOA, which prefer lower pH (Prosser and Nicol, 2012). This preference of AOA for low soil pH is attributed to their higher affinity for ammonia than AOB; at‍ ‍lower pH, the availability of ammonia decreases (NH_3_+H^+^↔NH_4_^+^; pKa=9.25), providing AOA with a competitive advantage over AOB (Prosser and Nicol, 2012; Wang *et al.*, 2019b). In contrast, although our Zambian soils were also nutrient-poor, average soil pH has increased with farming (Ohigashi *et al.*, 2021). Therefore, increased opportunities for ammonia availability due to fertilization and the increase in pH due to farming may both provide selective advantages to AOB in farmed soils in Zambia. However, the total abundance of AOB did not positively correlate with nitrification potential, while soil pH and several clusters showed positive correlations (Fig. S3). This result indicates that field soil pH or internal changes in the community of ammonia oxidizers may be crucial for nitrification potential, alongside the overall abundance of these microbes.

Several challenges have been associated with the interpretation of nitrification potential (Hazard *et al.*, 2021). One key issue is that measuring nitrification potential and investigating nitrifying microbes under a single laboratory condition may only partially reflect the actual state in the field. Moreover, the optimal conditions for nitrification differ among nitrifying species, making it difficult to capture the full spectrum of microbial activity in the laboratory (Webster *et al.*, 2005; Tourna *et al.*, 2010). We adjusted the‍ ‍pH to 7.2 when measuring nitrification potential and analyzing ammonia oxidizer communities, which raises the question of whether this approach accurately represents field conditions. However, we found that the community structure of prokaryotes in the slurry after a 1-h incubation was nearly identical to that in the field soil (Fig. S4A). Furthermore, it retained key features, such as greater Shannon diversity, more in farmlands than in natural lands (Fig. S4B), which is consistent with the results from field soil (Ohigashi *et al.*, 2021). This suggests that our approach mostly reflects the field state. Additionally, no significant changes were observed in the community structures of ammonia oxidizers between the 1-h and end-of-incubation groups (PERMANOVA, *P*>0.05), indicating that no specific ammonia oxidizers disproportionately grew and contributed to nitrification during the incubation, at least statistically (Fig. S5). Moreover, we acknowledge that the incubation condition at pH 7.2 may have provided an advantage for microbes at Site A, where field soil pH was closer to this level (Ohigashi *et al.*, 2021). The effects of community composition differences and environmental similarities on nitrification potential may not have been separated. Nevertheless, it is important to note that at Site B, despite the lack of a significant pH difference between natural land and farmland, farmland exhibited a significantly higher nitrification potential (Fig. 1). This suggests that factors beyond environmental similarities, such as the specific ammonia oxidizer community composition, have a crucial impact on nitrification potential. We addressed the challenges posed by differences between field and incubation conditions as well as concerns that incubation conditions may favor specific ammonia-oxidizing species.

In Zambia, AOB communities exhibited common and site-specific responses to land-use changes, while AOA communities showed robustness to land-use changes. The consistent relative increase in AOB cluster 3a.2 across all sites, which strongly correlated with nitrification potential, underscores the importance of targeted interventions, such as species-specific nitrification inhibitors (Nishigaya *et al.*, 2016). Additionally, site-specific changes were considered to be associated with inherent soil characteristics and farming management styles. This emphasizes the need for the further accumulation of detailed soil physicochemical data along with farming management information to predict localized responses and nitrification rates, particularly in regions such as Africa, where microbiome data is limited (Makhalanyane *et al.*, 2023). Furthermore, although the AOA community structure appeared robust against land-use changes, the total abundance of AOA decreased. This result indicates the need for a comprehensive evaluation of ecosystem functions rather than focusing solely on their microbial structural stability. These insights may lead to the selection of optimal farming practices, thereby enhancing nitrogen retention in sub-Saharan Africa.

## Conclusion

The present study revealed that nitrification potential was significantly higher in farmlands than in natural lands in Zambia. Land-use changes significantly affected the community structure of AOB. In farmlands, the relative abundance of cluster 3a.2 increased, possibly due to higher soil pH. Additionally, site-specific effects of land-use changes were observed; the Nsp58 lineage was abundant in the farmland at one site, while cluster 3b became abundant in the farmlands at sites with an originally low pH and high ammonia content. In contrast, the community structure of AOA was more strongly affected by site-specific characteristics, such as the original soil pH, as represented by the dominance of cluster NS-ζ in one site. However, the total abundance of AOA was lower in farmlands than in natural lands, indicating that AOA was generally vulnerable to farming regardless of the species. These results highlight the potential for broader-scale interventions, such as the development of nitrification inhibitors targeting a specific cluster or tailored pH adjustments, while also emphasizing the importance of considering site-specific responses and accumulating data across various locations that will contribute to the retention of soil nitrogen under agricultural practices in sub-Saharan Africa.

## Citation

Ohigashi, T., Mori, S., Tago, K., Ohbayashi, T., Hara, S., and Uchida, Y. (2025) Differential Responses of Soil Ammonia-oxidizing Bacterial and Archaeal Communities to Land-use Changes in Zambia. *Microbes Environ ***40**: ME24049.

https://doi.org/10.1264/jsme2.ME24049

## Supplementary Material

Supplementary Material
