# Supplementary material for: Differential Responses of Soil Ammonia-oxidizing Bacterial and Archaeal Communities to Land-use Changes in Zambia
Source: Microbes Environ. 2025 Mar 14;40(1):ME24049. doi: 10.1264/jsme2.ME24049 (PMC11946413; doi:10.1264/jsme2.ME24049)
Supplement: Supplementary file 1 — Supplementary Material [file 40_24049_s1.pdf]

# Supplementary Information for "Differential Responses of Soil Ammonia-oxidizing Bacterial and Archaeal Communities to Land-use Changes in Zambia"

Takamitsu Ohigashi, Suzumi Mori, Kanako Tago, Tsubasa Ohbayashi, Shintaro Hara, Yoshitaka Uchida

## Contents:


- **Supplementary Method 1.** Comparison of prokaryotic communities between the environment and the slurry under incubation
- **Supplementary Method 2.** Comparison of AOB and AOA communities between one-hour and end-of-incubation slurry samples
- **Figure S1.** Soil inorganic nitrogen
- **Figure S2.** Relative abundances of ammonia oxidizers
- **Figure S3.** Spearman's correlations between soil physicochemical properties
- **Figure S4.** States of the prokaryotic community under incubation
- **Figure S5.** Community structures of AOB and AOA throughout the incubation
- **Table S1.** Farming management in the studied sites
- **Table S2.** Slurry-sampling timings during the incubation for inorganic nitrogen content measurements
- **Table S3.** Number of filtered, denoised, and chimera-removed reads
- **Table S4.** Copy numbers of AOB and AOA in soils in other regions

**Supplementary Method 1. Comparison of prokaryotic communities between the environment and the slurry under incubation**

This analysis was conducted to compare the microbial communities in the incubation with those in the environment, which was demonstrated in Ohigashi et al. (2021) for the same soils. The extracted DNA samples from the slurry sampled at one hour after starting the incubation were amplified by PCR of the 16S rRNA gene. The forward primer 515f (5'-GTGCCAGCMGCCGCGGTAA-3') labeled with the Ion Xpress Barcode Adapters Kit (Thermo Fisher Scientific K.K., Yokohama City, Japan) and the reverse primer 806r (5'-GGACTACVSGGGTATCTAAT-3') labeled with the Ion P1 adapter (Thermo Fisher Scientific) were used. The mixture containing 1 µL of extracted DNA, 10 µL of Amplitaq Gold Master Mix, 0.4 µL of each primer, and 8.2 µL of nuclease-free water was amplified under the following conditions: 95°C for 10 min, followed by 30 cycles of 95°C for 30 s, 57°C for 30 s, and 72°C for 1 min. All obtained PCR products were then purified with AMPure XP (Beckman Coulter, CA, USA) following the manufacturer's protocol. The final length of the amplicons was checked by the Bioanalyzer 2100 (Agilent) using Bioanalyzer High Sensitivity DNA Kit (Agilent) according to the manufacturer's protocol. The libraries were then diluted to 50 pM with nuclease-free water. The Ion Chef Instrument (Thermo Fisher Scientific) with the Ion PGM Hi-Q Chef kit was used to load the library into the Ion 318 chip (Thermo Fisher Scientific). DNA sequencing was performed on the Ion PGM Sequencer (Thermo Fisher Scientific) with Ion PGM 400 Kits.

In Ohigashi et al. (2021), three cores were collected from the environment at each plot. For our current study, we used the third core (C3) for incubation experiments. To compare the microbial communities between the environmental and incubation samples, we downloaded the sequence data corresponding to the third core from each plot. Therefore, we specifically retrieved these data in FASTQ format from NCBI SRA (accession: PRJNA664260). The sequence data from the environmental and incubation samples was processed using the dada2 package on R software for adapter trimming, quality filtering, denoising, chimera removal, and the determination of amplicon sequencing variants (ASVs) as described in the main article. The sampling depth for random subsampling to equalize sample sizes was set to the minimum number of non-chimeric sequences across samples. From the sequences, we obtained 6605 ASVs. The Shannon diversity index was calculated for each sample using the diversity function. A two-way analysis of variance (ANOVA) was then conducted on the Shannon diversity index of the incubation samples, with 'land use' and 'site' as the factors. The dissimilarity in the community structure of prokaryotes was analyzed through non-metric multidimensional scaling (NMDS), employing the Bray-Curtis dissimilarity index using metaMDS function. Permutational multivariate analysis of variance (PERMANOVA) was employed to assess the differences in the community structures. This analysis considered the factors of ‘land use’ (Farm = 1, Natural = 2), ‘site’ (A = 1, B = 2, C = 3), and ‘source’ (Environment = 1, Slurry = 2). The adonis function from the vegan package was used for this analysis.

**Supplementary Method 2. Comparison of AOB and AOA communities between one-hour and end-of-incubation slurry samples**

We compared the communities of ammonia-oxidizing bacteria (AOB) and ammonia-oxidizing archaea (AOA) between slurry samples obtained at one hour and at the end of the incubation period. The analysis focused on assessing whether significant shifts occurred in the community structures during the incubation, which could indicate changes in the active ammonia-oxidizing populations.

The data for the one-hour incubation groups are the same as those presented in the main article. For the end-of-incubation groups, DNA sequencing, sequence processing, and BLAST searches were conducted using the same methods as described in the main article. To ensure consistent sample sizes, the sampling depths for random subsampling were conducted based on the smallest number of sequences identified in the BLAST results. Consequently, 134 ASVs for AOB and 25 ASVs for AOA were obtained. The dissimilarity in the community structure of prokaryotes was analyzed through NMDS, employing the Bray-Curtis dissimilarity index using metaMDS function. To assess the differences in the community structures, PERMANOVA was employed. This analysis considered the factors of ‘land use’ (Farm = 1, Natural = 2), ‘site’ (A = 1, B = 2, C = 3), and ‘timing’ (one-hour incubation = 1, end-of-incubation = 2) using the adonis function from the vegan package.

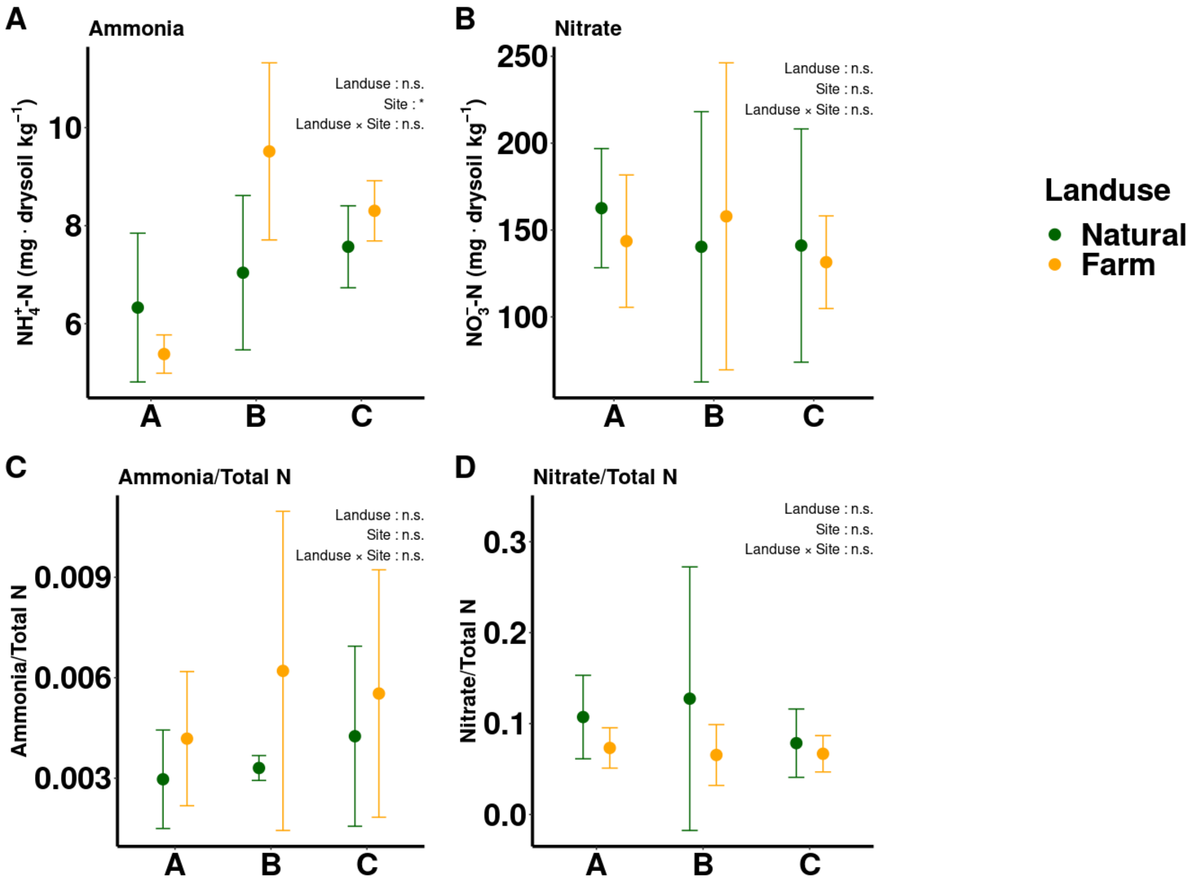


**Figure S1. Soil inorganic nitrogen.** The mean of (A) Ammonium-N and (B) Nitrate-N contents of the soils and (C, D) their ratio to total N are shown as the points. The error bars represent the standard deviation. For statistics, the data were analyzed with a two-way ANOVA for the site and land use. The significant differences in the effects were shown with p-values of *, **, or ***, representing $p < 0.05$, $p < 0.01$, or $p < 0.001$, respectively.

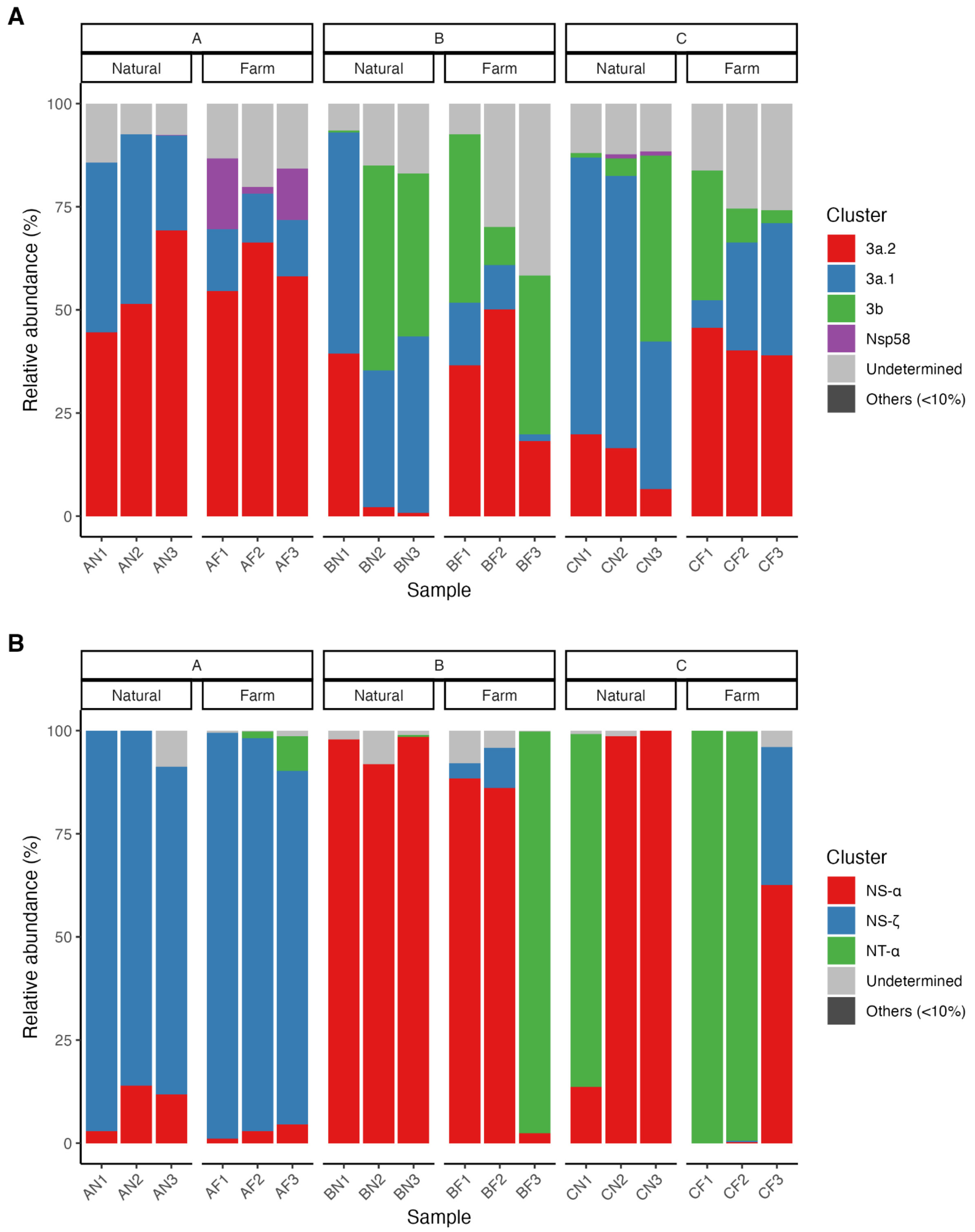


**Figure S2. Relative abundances of ammonia oxidizers.** Relative abundances of the clusters of (A) AOB and (B) AOA communities are shown. ASVs that were not phylogenetically close to reference sequences in the BLAST analysis were classified as "Undetermined."

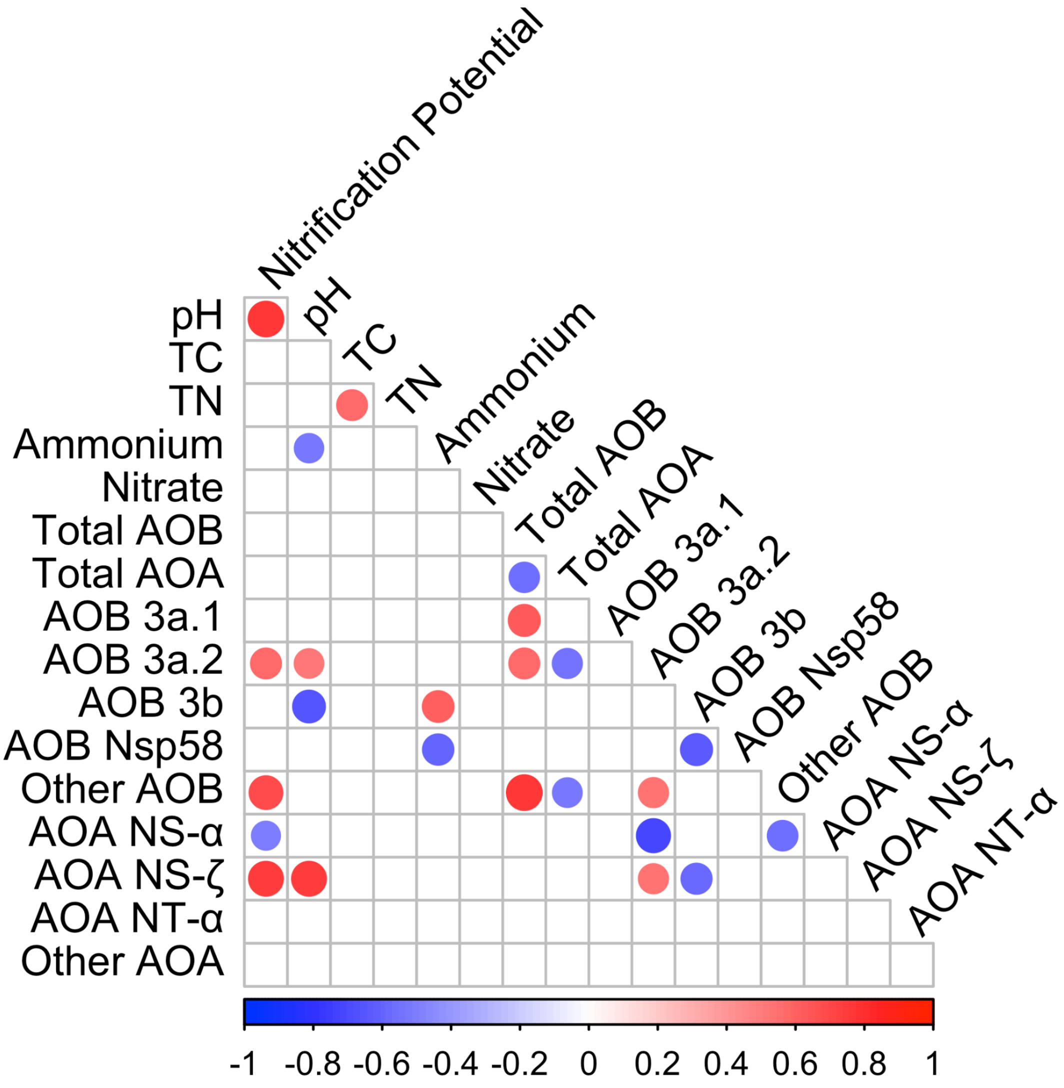


**Figure S3. Spearman's correlations between soil physicochemical properties.** The color and size of points indicate correlation coefficients; blue shows negative correlations while red shows positive correlations. Points are plotted if the pair correlates significantly ($p < 0.05$).

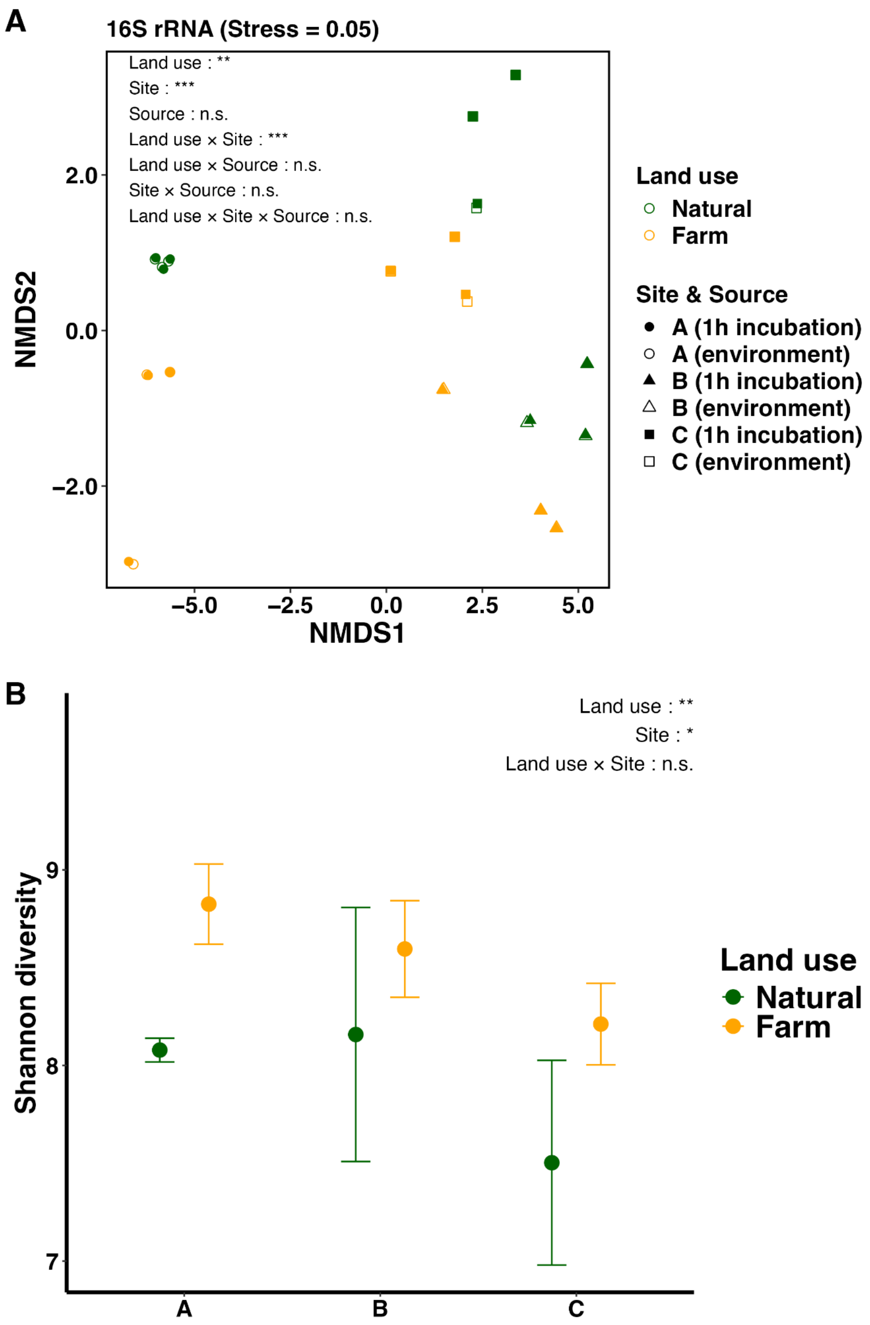


**Figure S4. States of the prokaryotic community under incubation.** (A) NMDS plot for prokaryotic communities in the slurry sampled at one hour after starting the incubation (1h incubation), and in the field soil which was investigated in Ohigashi et al., 2021 (environment). The effects of site, land-use change, and source of the DNA were tested with PERMANOVA. (B) Shannon diversity index of the prokaryotic communities in the slurry sampled at one hour after starting the incubation. The indices were analyzed with a two-way ANOVA for the site and land use. The significant differences in the effects were shown with p-values of *, **, or ***, representing $p < 0.05$, $p < 0.01$, or $p < 0.001$, respectively.

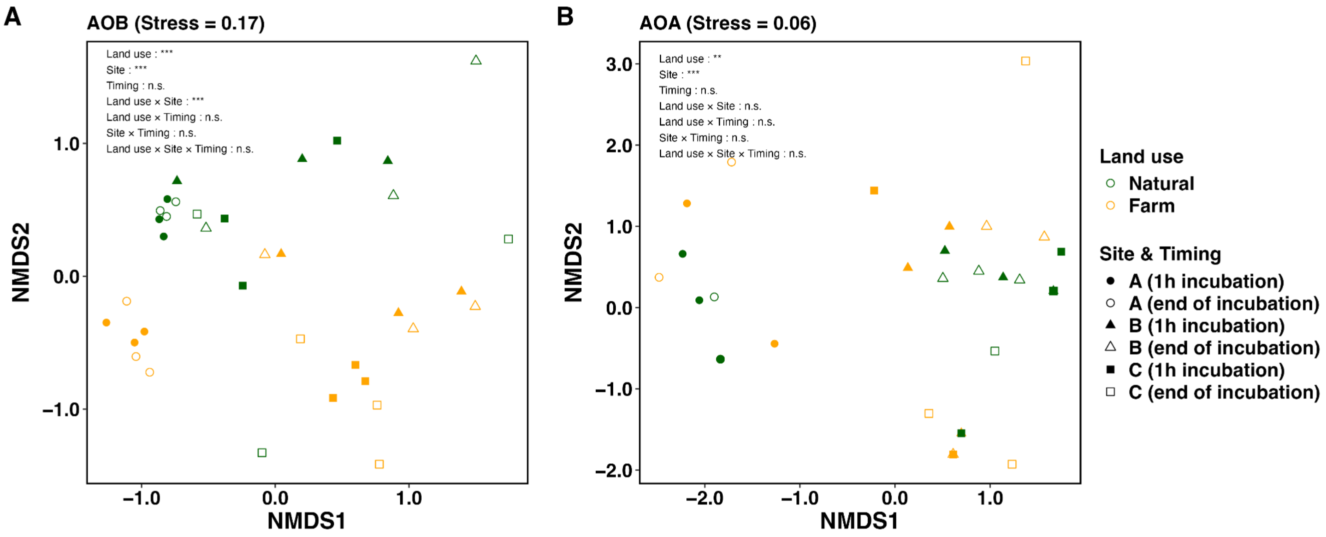


**Figure S5. Community structures of AOB and AOA throughout the incubation.** The community structures of (A) AOB and (B) AOA in the slurries that were sampled at one hour after starting the incubation (1h incubation) and were sampled at the end of incubation (end of incubation). The effects of site, land-use change, and sampling timing during the incubation were tested with PERMANOVA. The significant differences in the effects were shown with p-values of *, **, or ***, representing $p < 0.05$, $p < 0.01$, or $p < 0.001$, respectively.
